# Inhibition of SIK2 and SIK3 during differentiation enhances the anti-inflammatory phenotype of macrophages

**DOI:** 10.1042/BCJ20160646

**Published:** 2017-02-03

**Authors:** Nicola J. Darling, Rachel Toth, J. Simon C. Arthur, Kristopher Clark

**Affiliations:** 1 MRC Protein Phosphorylation and Ubiquitylation Unit, University of Dundee, Sir James Black Centre, Dow Street, Dundee DD1 5EH, U.K.; 2 Division of Cell Signalling and Immunology, School of Life Sciences, University of Dundee, Sir James Black Centre, Dow Street, Dundee DD1 5EH, U.K.

**Keywords:** HG-9-91-01, IL-10, inflammation, macrophage, salt-inducible kinase, TNF-α

## Abstract

The salt-inducible kinases (SIKs) control a novel molecular switch regulating macrophage polarization. Pharmacological inhibition of the SIKs induces a macrophage phenotype characterized by the secretion of high levels of anti-inflammatory cytokines, including interleukin (IL)-10, and the secretion of very low levels of pro-inflammatory cytokines, such as tumour necrosis factor α. The SIKs, therefore, represent attractive new drug targets for the treatment of macrophage-driven diseases, but which of the three isoforms, SIK1, SIK2 or SIK3, would be appropriate to target remains unknown. To address this question, we developed knock-in (KI) mice for SIK1, SIK2 and SIK3, in which we introduced a mutation that renders the enzymes catalytically inactive. Characterization of primary macrophages from the single and double KI mice established that all three SIK isoforms, and in particular SIK2 and SIK3, contribute to macrophage polarization. Moreover, we discovered that inhibition of SIK2 and SIK3 during macrophage differentiation greatly enhanced the production of IL-10 compared with their inhibition in mature macrophages. Interestingly, macrophages differentiated in the presence of SIK inhibitors, MRT199665 and HG-9-91-01, still produced very large amounts of IL-10, but very low levels of pro-inflammatory cytokines, even after the SIKs had been reactivated by removal of the drugs. Our data highlight an integral role for SIK2 and SIK3 in innate immunity by preventing the differentiation of macrophages into a potent and stable anti-inflammatory phenotype.

## Introduction

Macrophages play diverse roles in the immune response due to their innate ability to adapt their physiology to the changing needs of the host [1–3]. In response to infection or tissue damage, macrophages acquire an inflammatory phenotype characterized by the production of high levels of pro-inflammatory cytokines, such as tumour necrosis factor α (TNF-α), interleukin (IL)-12 and IL-1, a state often referred to as ‘classically activated’ or M1 macrophages. This initial event triggers a cascade of processes to establish an immune response to clear the infection and help repair tissue damage. Upon completion, inflammation must be resolved. To this end, macrophages switch to a pro-resolution phenotype characterized by the production of high levels of anti-inflammatory cytokines, including IL-10 and IL-1 receptor antagonist (IL-1ra) [1,4]. Various factors, including apoptotic neutrophils, immune complexes, IL-10 and prostaglandin E_2_ (PGE_2_), have been shown to promote diverse but related anti-inflammatory, pro-resolution phenotypes in macrophages, which have previously been grouped into M2 subsets [1,5]. The capacity of these subsets to modulate the immune response *in vivo* was demonstrated by showing that injection of these macrophage populations could protect mice from endotoxic shock [6]. Since the persistent presence of inflammatory macrophages is a feature of several human diseases, including rheumatoid arthritis and atherosclerosis [7–9], understanding the signalling pathways controlling the switch from inflammatory M1 to pro-resolution M2-like macrophages may identify new therapeutic strategies for the treatment of these diseases.

Macrophage polarization to inflammatory or anti-inflammatory, pro-resolution states involves two signals: the first signal activates the transcriptional programme encoding both the pro-inflammatory and anti-inflammatory mediators; the second signal reinforces either the classically activated, M1 phenotype or the anti-inflammatory, pro-resolution M2-like phenotype [1]. Ligation of Toll-like receptors (TLRs) triggers a signalling platform leading to the activation of core transcriptional factors, including nuclear factor κB (NF-κB) and interferon regulatory factors (IRF3/IRF5), for the production of pro-inflammatory cytokines, while cyclic AMP (cAMP) response element-binding protein (CREB) induces the transcription of anti-inflammatory genes, including IL-10, dual specificity phosphatase (DUSP) 1 and Nur77 [10]. It is the balance in the activities of the different transcriptional factors that dictates the overall phenotype of the macrophage. One mechanism by which the second signal can influence this balance, and thereby macrophage polarization, is by affecting the transcriptional output from CREB. For example, interferon γ (IFN-γ) promotes the inflammatory M1 phenotype by interfering with CREB function to suppress the production of IL-10 [11], whereas cAMP-elevating agonists, such as PGE_2_, drive regulatory macrophages by activating CREB to induce substantial production of IL-10 [12].

CREB function is regulated in macrophages by two major signalling mechanisms. The protein kinases, such as mitogen- and stress-activated protein kinase (MSK) 1/2, phosphorylate CREB at Ser133 in response to TLR stimulation [13]. This results in the transcriptional activation of CREB [14] and consequent induction of IL-10. The activity of CREB can be further enhanced through interactions with co-activators, such as the CREB-regulated transcription co-activator (CRTC) family [15]. Under basal conditions, CRTCs are phosphorylated by members of the AMP-activated protein kinase-related kinase family, which creates binding sites for 14-3-3 proteins [16]. The CRTC–14-3-3 complexes are retained in the cytosol, thereby keeping CREB activity low. Stimuli that promote the dephosphorylation of CRTCs induce the dissociation of CRTCs from 14-3-3, which facilitates their translocation into the nucleus where they interact with CREB. We found that the salt-inducible kinases (SIKs) suppress IL-10 production by phosphorylating CRTC3 in macrophages [17]. Pharmacological inhibition of the SIKs promoted the dephosphorylation of CRTC3 at Ser62, Ser162, Ser329 and Ser370, which rapidly migrated into the nucleus to elevate CREB-dependent gene transcription including that of IL-10, in both mouse and human macrophages [17]. We further demonstrated that cAMP-elevating stimuli, including small-molecule inhibitors of phosphodiesterases and the physiological agonist PGE_2_, also induce IL-10 production via a protein kinase A-dependent signalling pathway that interferes with the ability of the SIKs to phosphorylate CRTC3 [12]. Thus, the MSKs and SIKs play key roles in defining CREB-dependent gene transcription in macrophages, including the production of IL-10.

Pro-resolution M2-like macrophages are also defined by the production of low levels of pro-inflammatory cytokines, including TNF-α and IL-12p40, and can be distinguished from other macrophage populations by the expression of increased levels of arginase 1 (Arg1), sphingosine kinase 1 (SPHK1) and TNF ligand superfamily member 14 (LIGHT) mRNA [1,4]. Importantly, inhibition of the SIKs promotes all of these features in macrophages, including the suppression of TNF-α, IL-12p40 and IL-6 secretion [12,17]. These pro-inflammatory cytokines are all controlled by the transcription factor NF-κB. TLR stimulation activates NF-κB through the interplay between phosphorylation and ubiquitylation events [18]. However, p65 is also acetylated at Lys310, which is a mechanism for fine-tuning the activity of NF-κB [19]. Since the SIKs regulate the nuclear shuttling of class IIA histone deacetylases (HDACs) in skeletal myotubes [20], it has been proposed that one mechanism by which these kinases promote the production of pro-inflammatory cytokines is through the phosphorylation and retention of HDAC4 in the cytosol [21]. Stimulation of macrophages with cAMP-elevating agonists interferes with the ability of the SIKs to phosphorylate HDAC4, leading to its accumulation in the nucleus where it can promote the deacetylation of p65 at Lys310 and thereby its release from the promoter region of TNF-α and IL-12p40 [21]. The SIKs therefore represent a novel molecular switch controlling macrophage polarization by balancing the activities of CREB and NF-κB, but these kinases most likely impinge on macrophage fate by additional mechanisms that remain to be discovered.

Taken together, the data suggest that the SIKs are attractive targets for the development of new anti-inflammatory drugs. Interestingly, we and others discovered that several inhibitors of protein tyrosine kinases that have been approved as anti-cancer drugs also potently inhibit the SIKs, which provides an indication that specific and potent SIK inhibitors will be tolerated in humans [22,23]. There are three isoforms of the SIKs, namely SIK1, SIK2 and SIK3, but the role of each kinase in macrophage biology remains elusive, largely because all of the small-molecule inhibitors identified so far do not discriminate between the different isoforms [17,22,23]. Here, we combined mouse genetics, chemical biology and enzymology to address this question.

## Materials and methods

### Materials

The small-molecule kinase inhibitors, HG-9-91-01 and MRT199665, were synthesized as previously described [17]. The pharmacological inhibitors were dissolved in DMSO and stored as 10 mM solutions at −20°C. Mouse macrophage colony-stimulating factor (M-CSF) and IFN-γ were purchased from Peprotech, lipopolysaccharide (LPS) (*Escherichia coli* strain O5:B55) was obtained from Alexis Biochemicals and Pam_3_CSK_4_ was acquired from Invivogen.

### Generation of KI mice

SIK1-T182A, SIK2-T175A and SIK3-T163A kinase dead knock-in (KI) mutations were created by conventional gene targeting technologies by TaconicArtemis. Briefly, vectors were created to introduce the desired mutations in the SIK1, 2 or 3 genes via homologous recombination in embryonic stem (ES) cells (Supplementary Figure S1A–C). At the same time, loxP sites were introduced 3′ and 5′ to the targeted exons. Vector sequences are available on request. Targeting was carried out in Art B6 3.6 (genetic background: C57Bl/6 N Tac) ES cells and the correctly targeted clones confirmed by Southern blotting using 5′ and 3′ probes that bound to sites outside the sequences used in the targeting vector (Supplementary Figure S1A–C). The presence of the desired mutation was further confirmed by PCR of the appropriate genomic region followed by sequencing (data not shown). ES cells were then used to create chimeric mice using standard methods. Chimeric mice were bred to *Flpe* transgenic mice, which in the case of germline transmission would also result in the deletion of the Frt or F3 flanked markers used for selection in the ES cell targeting. From these crosses, mice heterozygous for the desired mutation were detected by PCR genotyping analysis and subsequently crossed to remove the *Flpe* transgene. Routine genotyping for the mutated alleles was carried out by PCR using the primers CTTGCTCTTAAGGTGTTCTTAAGG and ACAGAGCCACTAACAAGACTGC for SIK1, AAGAGAGTGTGGGACTAACTTGG and GCTTGTTTACAGAGTGCAGTGG for SIK2 or GGCTAGTTAGAGAAATGCACAGG and CATGTGCACCTGGAAAAGG for SIK3. Mice were maintained on a C57Bl/6 background and provided with free access to food and water. Animals were kept in ventilated cages under specific pathogen-free conditions in accordance with UK and EU regulations. Experiments were carried out subject to local ethical review under a UK Home Office project license.

### DNA constructs

Vectors to express human SIK1 (DU1103), SIK2 (DU53066) and SIK3 (DU46915) with an N-terminal HA-tag in mammalian cells were generated by amplifying the relevant cDNA fragments by PCR, ligating the product into the pCR2.1 TOPO vector (Invitrogen), sequencing the insert and then subcloning the fragment into pCMV5. PCRs were carried out using KOD Hot Start DNA polymerase (Novagen), and DNA sequencing was performed by the MRC-PPU Sequencing Service (https://www.dnaseq.co.uk). The coding region of human SIK1 cDNA (NCBI Acc. NM_173354) was amplified from IMAGE consortium EST clone 4831049 (NCBI Acc. NM_173354) using primers 5′-GCGAATTCGCCACCATGTACCCATACGATGTGCCAGATTACGCCGTTATCATGTCGGAGTTCAGCGCGG-3′ and 5′-GCGGATCCTCACTGCACCAGGACAAACGTGCC-3′, and subcloned as an *Eco*RI/*Bam*HI insert into pCMV5; the coding region of human SIK2 cDNA (NCBI Acc. XM_041314) was amplified from IMAGE consortium EST clone 5495545 (NCBI Acc. BM799630) using primers 5′-GCGGTACCGCCACCATGTACCCATACGATGTGCCAGATTACGCCGTCATGGCGGATGGCC CGAG-3′ and 5′-GCGTCGACTAATTCACCAGGACATACCCGTTGTG-3′, and subcloned as a *Kpn*I/*Sal*I insert into pCMV5; full-length SIK3 was re-amplified from the pGEX6P-SIK3 construct previously generated [24] using primers 5′-GCGAATTCGCCACCATGTACCCATACGATGTGCCAGATTACGCCGCGGCGGCGGCGGCGAGCGG-3′ and 5′-GCGAATTCGGCTTTTACACGCCTGCCTGCTCC-3′, and subcloned as an *Eco*RI/*Eco*RI insert into pCMV5. All plasmids are available through MRC-PPU Reagents and Services (https://mrcppureagents.dundee.ac.uk).

### Antibodies

Antibodies were raised against peptides encompassing amino acids 577–592 or 597–612 of human SIK1 (S306D, bleed 3), amino acids 272–445 of mouse SIK2 (S567D, bleed 3) and amino acids 926–1038 of mouse SIK3 (S373D, bleed 3) in sheep and affinity purified by the antibody production team at MRC-PPU Reagents and Services (https://mrcppureagents.dundee.ac.uk). The following antibodies were used for immunoblotting: horseradish peroxidase-conjugated secondary antibodies (Pierce), anti-HA (Roche), anti-CRTC3 (Abcam), anti-glyceraldehyde 3-phosphate dehydrogenase (GAPDH), anti-nitric oxide synthase (NOS), anti-SIK2, anti-signal transducer and activator of transcription (STAT) 3, anti-pY705-STAT3 and anti-pS246-HDAC4/pS259-HDAC5/pS155-HDAC7 (Cell Signalling Technology).

### Cell culture

Primary macrophages were generated by differentiating bone marrow from 6- to 12-week-old C57Bl/6 mice or foetal livers from day E14.5–E15.5 embryos for 7–10 days in DMEM supplemented with 10 ng/ml purified recombinant M-CSF or 20% (v/v) L929 conditioned medium as a source of M-CSF, 2 mM glutamine, 10% (v/v) foetal calf serum (FCS), penicillin and streptomycin. Macrophages were differentiated on non-tissue culture-treated plastic, harvested and replated at a density of 100 000 cells/cm^2^/0.1 ml on tissue culture-treated plastic in fresh media prior to stimulation on day 8. Where macrophages were differentiated in the presence of inhibitors, these were added to the culture medium in 0.1% DMSO on days 0, 2 and 4. HEK293 cells were cultured in DMEM containing 10% (v/v) FCS, 2 mM glutamine and antibiotics, and were transfected using Lipofectamine 2000 (Invitrogen) following the manufacturers' instructions.

### Quantitative PCR

mRNA was extracted from cells using the MicroElute Total RNA (Omega Bio-Tek) or RNeasy micro (Qiagen) kit following the manufacturers' instructions. cDNA was generated from 0.5 µg of total RNA using the iScript cDNA synthesis kit and quantified by qPCR using the SsoFast EvaGreen Supermix on a CFX384 real-time system (Bio-Rad Laboratories). The relative expression of each gene was calculated from *C*_t_ values using the Pfaffl method [25] and was normalized against the mRNA levels of house-keeping genes, 18S and GAPDH. Results are reported relative to untreated control cells, which were set to 1. The following qPCR primers were used: 18S F, gtaacccgttgaaccccatt; 18S R, ccatccaatcggtagtagcg; Arg1 F, ctccaagccaaagtccttagag; Arg1 R, aggagctgtcattagggacatc; GAPDH F, gccttccgtgttcctaccc; GAPDH R, tgcctgcttcaccaccttc; IL-6 F, ttccatccagttgccttcttg; IL-6 R, aggtctgttgggagtggtatc; IL-10 F, ccctttgctatggtgtcctttc; IL-10 R, gatctccctggtttctcttccc; inducible NOS (iNOS) F, acatcgacccgtccacagtat; iNOS R, cagaggggtaggcttgtctc; LIGHT F, ctgcatcaacgtcttggaga; LIGHT R, gatacgtcaagcccctcaag; Nur77 F, cctgttgctagagtctgccttc; Nur77 R, caatccaatcaccaaagccacg; SPHK1 F, acagcagtgtgcagttgatga; SPHK1 R, ggcagtcatgtccggtgatg. PCRs generated a single product (as visualized on gel and melting curve) of the expected sequence (data not shown).

### Cytokine secretion

The concentrations of TNF-α, IL-6, IL-10 and IL-12p40 in cell-free culture supernatants were measured using the Bio-Plex Pro Assay system (Bio-Rad Laboratories). IL-10 was also measured using an ELISA kit from Peprotech.

### Immunoblotting

Tissues and cells were extracted in lysis buffer [50 mM Tris/HCl (pH 7.4), 1 mM EDTA, 1 mM EGTA, 50 mM sodium fluoride, 5 mM sodium pyrophosphate, 10 mM sodium β-glycerol 1-phosphate, 1 mM dithiothreitol, 1 mM sodium orthovanadate, 0.27 M sucrose, 1% (v/v) Triton X-100, 1 µg/ml aprotinin, 1 µg/ml leupeptin and 1 mM phenylmethylsulphonyl fluoride]. Cell extracts were clarified by centrifugation, and protein concentrations were determined using the Bradford assay. To detect proteins in cell lysates, 15–20 µg of protein extract was separated by SDS–PAGE. After transfer to PVDF membranes, proteins were detected by immunoblotting and visualized by treating the blots with enhanced chemiluminescence (Amersham) followed by autoradiography. Where indicated, the expression of SIKs in tissue extracts was visualized using the Li-cor Odyssey Fc imaging system and quantified using ImageStudio v4.

### Kinase assays

Endogenous SIK1, SIK2 and SIK3 were immunoprecipitated by incubating 1–8 mg of lysate with 1–4 µg of antibody raised against the indicated SIK sequence: SIK1, 577–592 or 597–612 human (S306D, bleed 3); SIK2, 272–445 mouse (S567D, bleed 3); SIK3, 926–1038 mouse (S373D, bleed 3), for 1 h at 4°C prior to the addition of 5 µl of Protein G-Agarose beads and incubation for a further hour at 4°C. Immunoprecipitates were washed three times with lysis buffer containing 0.5 M sodium chloride and twice with assay buffer [50 mM Tris/HCl (pH 7.4), 0.1 mM EGTA, 10 mM magnesium acetate and 2 mM dithiothreitol]. Kinase assays were initiated by incubating immunoprecipitated protein with 0.1 mM [γ-^32^P]-ATP (300 cpm/pmol) and 300 µM substrate peptide ALNRTSSDSALHRRR (corresponding to human CRTC2 residues 165–176 followed by three arginine residues to aid binding to phosphocellulose paper) for 20 min at 30°C. Incorporation of ^32^P-phosphate into the substrate peptide was determined by applying the reaction to p81 phosphocellulose paper and scintillation counting after washing with 50 mM othophosphoric acid and fixing in acetone. Data were converted into Units/mg of protein extract where 1 Unit is equivalent to the amount of enzyme required to incorporate 1 nmol of ^32^P into the substrate per minute.

### Flow cytometry

Naive macrophages were detached from non-tissue culture-treated plates after 7 days differentiation in M-CSF-containing media by scraping in versene. Cells were pelleted (1000 ***g***, room temperature, 5 min), washed in FACS buffer [1% (w/v) bovine serum albumin in phosphate buffered saline (PBS)] and then kept at 4°C for subsequent steps. Cells were resuspended and incubated with 10 µg/ml anti-CD16/32 Fc block (BD Bioscience) in FACS buffer for 15 min, prior to incubation for 30 min with 1 µg/ml Brilliant Violet 421 anti-mouse F4/80 and 1 µg/ml PerCP/Cy5.5 anti-mouse/human CD11b (Biolegend) in FACS buffer. Cells were washed in FACS buffer and fixed by incubation with 4% formaldehyde in PBS for 20 min. Fixed cells were washed and resuspended in FACS buffer prior to analysis using FACS Canto (Becton Dickinson).

### Cytokine levels in mouse serum

SIK2 KI mice on a C57Bl/6 background and wild-type (WT) littermates (8–12 weeks old) were injected intraperitoneally with LPS (1.8 mg/kg). One hour later, the mice were euthanized and the concentrations of cytokines in the sera were measured using the Bio-Plex Pro Assay System (Bio-Rad Laboratories).

### Nitric oxide assay

Nitrite levels in cell-free culture supernatants were assayed using the Griess reaction in samples treated with or without nitrate reductase and nicotinamide adenine dinucleotide. Standards and reagents were supplied by R&D Systems. Nitrite and nitrate levels were normalized to protein concentration in cellular extracts.

### Statistical analysis

Quantitative data are presented as the mean ± SEM. In most experiments using primary macrophages, cultures were established from ‘*n*’ mice. Each macrophage population was independently differentiated, cultured and stimulated prior to analyzing the biological material. Where indicated, macrophages were pooled following differentiation. Statistical significance of differences between experimental groups was assessed using the one-way or two-way ANOVA with Bonferroni post-test or Student's unpaired *t*-test when appropriate. Unless otherwise indicated, the data are compared with cells stimulated with TLR agonists in the absence of any drug. Differences in means were considered significant if *P *< 0.05; *P < 0.05, **P < 0.01, ***P < 0.001. n.s. refers to non-significant.

## Results

### SIK2 is the major contributor to the overall activity of the SIKs in bone marrow-derived macrophages

**Figure 1.**
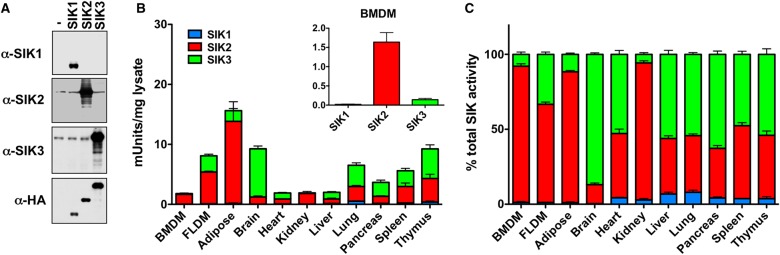
Enzymatic activity of different SIK isoforms in mouse macrophages and tissues. (**A**) Development of isoform selective antibodies against SIK1, SIK2 and SIK3. HEK293 cells were transfected with vectors encoding HA-SIK1, HA-SIK2, HA-SIK3 or the empty vector as a negative control. Cell extracts were separated by SDS–PAGE and immunoblotted with the indicated antibodies. In this instance, anti-SIK2 (S567D, bleed 3) was used for immunoblotting. (**B**) Kinase activity of SIK1, SIK2 and SIK3 in different mouse tissues reported in mUnits/mg of tissue extract. Kinase assays were performed as described in Materials and Methods (mean ± SEM, *n* = 3). (**C**) Contribution of each isoform to the total SIK activity in mouse tissues; data from (**B**).

To address the role of each isoform of the SIKs, we first set out to measure the enzymatic activity of SIK1, SIK2 and SIK3 in mouse macrophages. For this purpose, we raised a novel set of antibodies against SIK1, SIK2 and SIK3. Each antibody was selective for its respective antigen and did not detect the other family members as judged by immunoblotting extracts from cells overexpressing HA-tagged versions of human SIK1, SIK2 and SIK3 (Figure 1A). Moreover, the antibodies raised against SIK2 and SIK3 also detected the endogenous kinases (Figure 1A). All antibodies recognize both the human and mouse homologues of SIK1, SIK2 and SIK3 (data not shown).

Using these antibodies, we immunoprecipitated endogenous SIK1, SIK2 and SIK3, and determined the activity of each isoform in mouse foetal liver-derived macrophages (FLDMs) and bone marrow-derived macrophages (BMDMs) as well as various mouse tissues (Figure 1B). Notably, we detected enzymatic activity of SIK1, SIK2 and SIK3 in all mouse tissues tested in the present study (Figure 1B). Of the different isoforms, SIK2 had the highest level of activity in mouse macrophages (Figure 1B), contributing ∼90% of the total SIK activity towards the peptide substrate used in the present study, whereas SIK3 contributes ∼10% and SIK1 only ∼1% in BMDMs (Figure 1C). In all the cells and tissues studied, SIK1 was the least abundant isoform as judged by kinase activity. Adipose tissue, which expresses very high levels of SIK2 protein [26], showed the highest level of SIK activity (Figure 1B), whereas the levels of SIK2 kinase activity ranged from 1 to 5 mUnits/mg lysate across the other tissues. The enzymatic activity of SIK3 across mouse tissues varied considerably with BMDMs and kidney having low levels of activity, while the brain had the highest levels (Figure 1B). In terms of the percentage of total activity, SIK2 and SIK3 made similar contributions to the overall activity of the SIKs in most tissues (Figure 1C). Interestingly, the brain is unique among the tissues tested because SIK3 was the most abundant isoform while, in adipose tissue, kidney and macrophages, SIK2 made the greatest contribution to total SIK kinase activity (Figure 1C).

### Development of catalytically inactive KI mice of SIK1, SIK2 and SIK3

Consequently, we generated full-body KI mice of SIK1, SIK2 and SIK3 in which we replaced the WT protein with a catalytically inactive mutant following the strategy described in Supplementary Figure S1. We selected the threonine residue in the activation loop of the SIKs to mutate because the phosphorylation of this residue by LKB1 is essential for SIK catalytic activity [24]. The animals generated are SIK1-T182A, SIK2-T175A and SIK3-T163A. Homozygous SIK1 and SIK2 KI animals are viable and fertile, looking indistinguishable from WT littermates under standard housing conditions. In contrast, homozygous SIK3 KI animals genotyped at weaning (21–28 days old) are present at sub-Mendelian ratios (Supplementary Figure S2A,B). The SIK3 KI animals are, on average, smaller than their WT or heterozygous littermates (Supplementary Figure S2C,D). Pathological examination of the SIK3-T163A KI mice suggested defects in skeletal development that contribute to their smaller size (data not shown).

**Figure 2.**
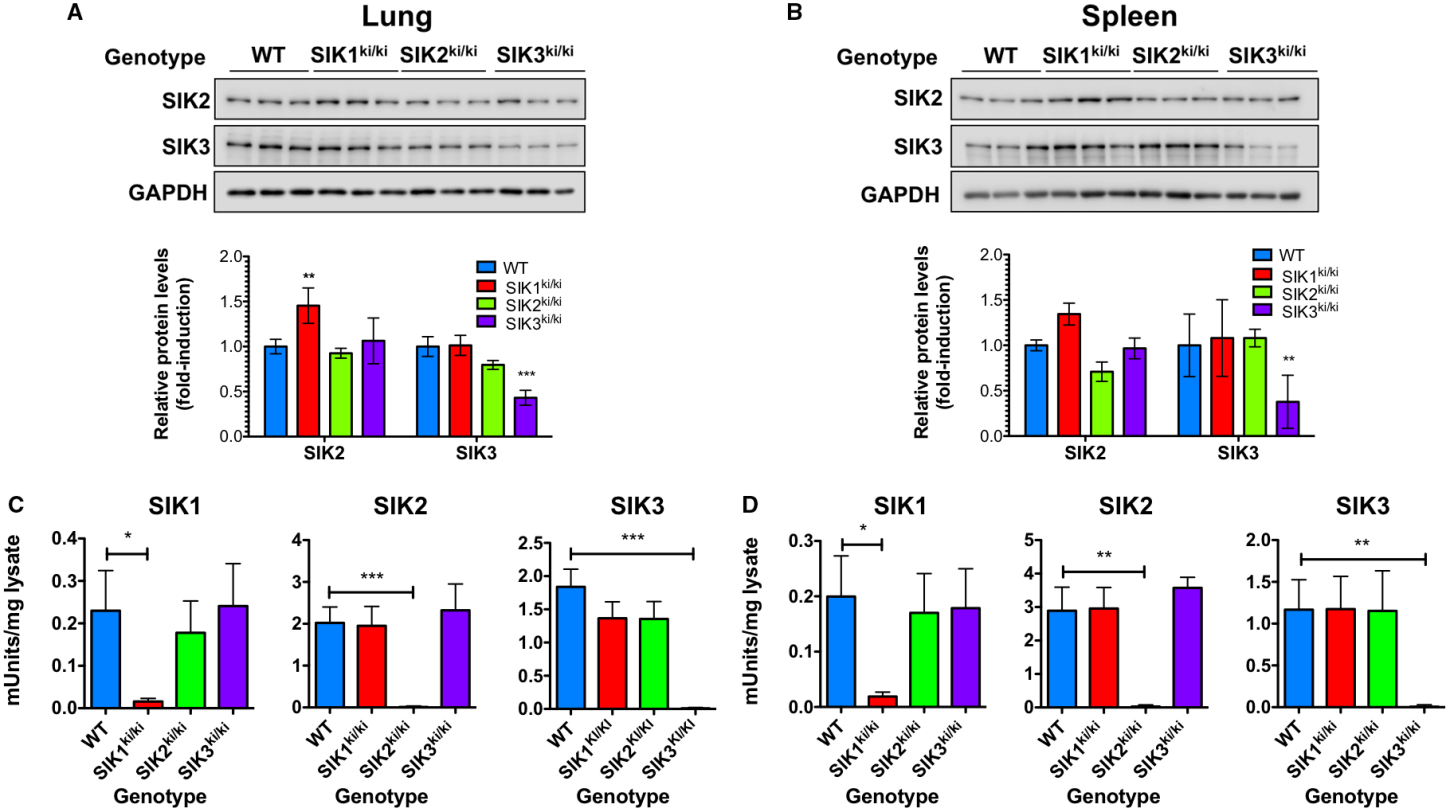
Generation of SIK1, SIK2 and SIK3 catalytically inactive KI mice. (**A** and **B**) Protein expression of SIK2 and SIK3 in (**A**) lung and (**B**) spleen tissue from the different mouse lines in comparison with WT littermate controls. Tissue extracts (20 µg) from three independent mice per genotype were separated by SDS–PAGE, immunoblotted using the indicated antibodies and visualized using the Li-cor Odyssey Fc imaging system. SIK protein levels were quantified relative to GAPDH (mean ± SD, *n* = 3), statistics represent analysis by two-way ANOVA and Bonferroni post-tests comparing each SIK genotype with WT. (**C** and **D**) Enzymatic activity of SIK1 (left panel), SIK2 (middle panel) and SIK3 (right panel) in the (**C**) lungs and (**D**) spleens from the different SIK KI mice in comparison with WT mice (mean ± SEM, *n* = 4). Statistics shown are the result of unpaired Student's *t*-tests.

To verify the loss of activity of endogenous SIKs carrying the alanine mutation in their activation loop, we measured the activity of SIK1, SIK2 and SIK3 from lung and spleen extracts obtained from each one of the KI animals and WT littermates. The expression of the SIK3-T163A protein in the tissues of the KI mice was slightly lower than that of the WT protein, whereas SIK2-T175A was expressed at similar levels to WT protein (Figure 2A,B). We were unable to identify any antibody capable of recognizing endogenous murine SIK1 by immunoblotting. Immunoprecipitation studies showed that the activities of SIK1, SIK2, and SIK3 were strongly reduced, if not completely ablated, only in the tissue extracts from the corresponding KI mice (Figure 2C,D). Thus, mutation of one isoform of the SIKs did not affect the overall expression or activity of the other two isoforms. These results indicate that the antibodies selectively immunoprecipitate the relevant isoform of the SIK against which it was raised and that endogenous SIK1, SIK2 and SIK3 do not hetero-oligomerize in mammalian cells.

### SIK2 has an important role in macrophage polarization

**Figure 3.**
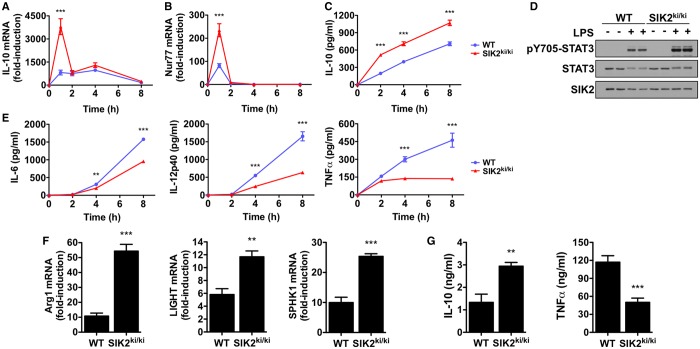
Macrophages from SIK2 KI mice have a pro-resolution M2-like phenotype. (**A** and **B**) Kinetics of (**A**) IL-10 and (**B**) Nur77 mRNA production in LPS-stimulated BMDMs from SIK2-T175A KI mice in comparison with WT controls. Macrophages were stimulated with 100 ng/ml LPS for the indicated times. mRNA levels were measured by qPCR and results are expressed as fold-induction relative to unstimulated WT cells (mean ± SEM, *n* = 4). (**C**) Kinetics of IL-10 secretion in LPS-stimulated BMDMs from SIK2 KI mice in comparison with WT controls. Experiment was performed as in (**A**), but the concentration of IL-10 was measured in culture supernatant using the Bio-Plex Pro Assay System (mean ± SEM, *n* = 4). (**D**) IL-10 signalling in SIK2 KI mice. BMDMs from WT and SIK2 KI mice were stimulated with 100 ng/ml LPS for 2 h and cell extracts were immunoblotted with the indicated antibodies. Results are representative of two independent experiments. (**E**) Reduced secretion of pro-inflammatory cytokines in LPS-stimulated BMDMs from SIK2 KI mice. Macrophages from WT and SIK2 KI mice were stimulated with 100 ng/ml LPS for the indicated times and cytokine levels in cell-free supernatants were measured using the Bio-Plex Pro Assay System (mean ± SEM, *n* = 4). (**F**) Enhanced expression of regulatory macrophage markers in cells derived from SIK2 KI mice. BMDMs from WT and SIK2 KI mice were stimulated with 100 ng/ml LPS for 4–8 h. mRNA levels of Arg1 (8 h), LIGHT (4 h) and SPHK1 (4 h) were measured by qPCR (mean ± SEM, *n* = 4). (**G**) Circulating IL-10 levels are increased, whereas TNF-α levels are decreased in SIK2 KI mice. Mice were administered LPS (1.8 mg/kg) by intraperitoneal injection and euthanized 1 h later. The concentration of IL-10 and TNF-α in mouse serum was measured using the Bio-Plex Pro Assay System (mean ± SEM, *n* = 4). Statistics in (**A–C** and **E**) represent analysis by two-way ANOVA and Bonferroni post-tests; statistics in (**F** and **G**) are the results of unpaired Student's *t*-test and compare SIK2 KI mice with WT.

Since SIK2 is the isoform with highest kinase activity in mouse macrophages, we initially investigated the role of this isoform in macrophage polarization. Stimulation of BMDMs from WT mice with LPS led to a low but sustained synthesis of IL-10 mRNA (Figure 3A). In contrast, BMDMs from SIK2-T175A KI mice display high and transient expression of IL-10 mRNA, peaking at 1 h and returning to levels seen in WT macrophages by 2 h (Figure 3A). A similar pattern of expression between WT and SIK2-T175A macrophages was observed for another CREB target gene Nur77 (Figure 3B), indicating that CREB-dependent gene expression was elevated in macrophages from SIK2-T175A mice. Consistent with higher mRNA levels of IL-10, macrophages from SIK2-T175A mice also secreted higher levels of IL-10 (Figure 3C), which led to increased phosphorylation of STAT3 (Figure 3D), a transcription factor activated by ligation of the IL-10 receptor [27]. In addition, macrophages from SIK2-T175A mice secreted lower levels of the pro-inflammatory cytokines IL-6, IL-12p40 and TNF-α in response to LPS in comparison with WT macrophages (Figure 3E). Finally, SIK2-T175A macrophages expressed higher levels of the markers Arg1, LIGHT and SPHK1 after LPS stimulation (Figure 3F). Our results indicate that SIK2 inhibition polarizes macrophages towards a pro-resolution M2-like phenotype.

Based on these results, we tested whether SIK2 activity also regulated cytokine production after LPS administration *in vivo*. Upon intraperitoneal injection of LPS into WT mice, there is a rapid (1 h) increase in circulating levels of IL-10 and TNF-α. Importantly, the circulating levels of IL-10 were higher while the levels of TNF-α were lower in SIK2-T175A mice administered LPS (Figure 3G). Thus, SIK2 controls cytokine production both *in vivo* and in isolated cells.

### All three SIK isoforms control macrophage polarization

**Figure 4.**
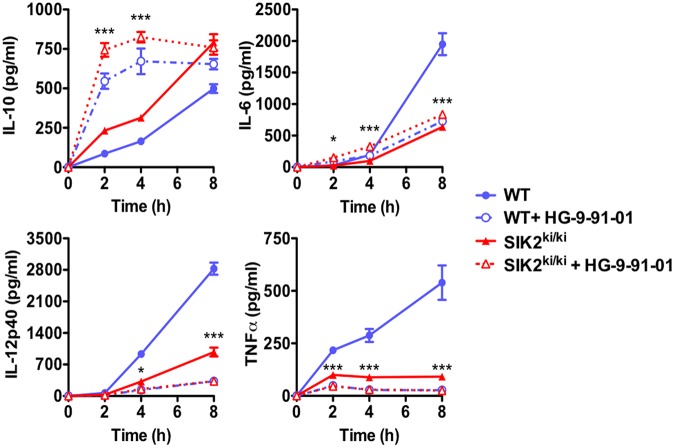
SIK2 and another isoform regulate macrophage polarization. BMDMs from WT (blue) and SIK2 KI mice (red) were treated with vehicle control (solid line) or 500 nM HG-9-91-01 (dashed line) for 1 h and then stimulated with 100 ng/ml LPS for the indicated times. The concentration of cytokines in cell-free culture supernatants was measured using the Bio-Plex Pro Assay System (mean ± SEM, *n* = 4). Statistics represent the results of two-way ANOVA and Bonferroni post-tests comparing SIK2 KI macrophages with and without HG-9-91-01 treatment.

If the inhibition of SIK2 is sufficient to induce the characteristic features of M2-like macrophages, then the addition of the pan-SIK inhibitor HG-9-91-01 to SIK2-T175A macrophages should have no further effect on cytokine production. Notably, the addition of HG-9-91-01 to SIK2-T175A macrophages further elevated IL-10 and further suppressed TNF-α and IL-12p40 production in response to LPS, ultimately reaching levels observed in WT macrophages treated with HG-9-91-01 (Figure 4). As seen previously, pan-SIK inhibition using HG-9-91-01 elevated IL-6 secretion at early time points in both WT and SIK2-T175A macrophages possibly due to CREB-dependent IL-6 transcription; but IL-6 secretion was suppressed following pan-SIK inhibition or SIK2 KI after stimulation with LPS for longer than 4 h (Figure 4 and Supplementary Figure S3) [12]. Thus, SIK1 and/or SIK3, in addition to SIK2, regulate macrophage polarization.

**Figure 5.**
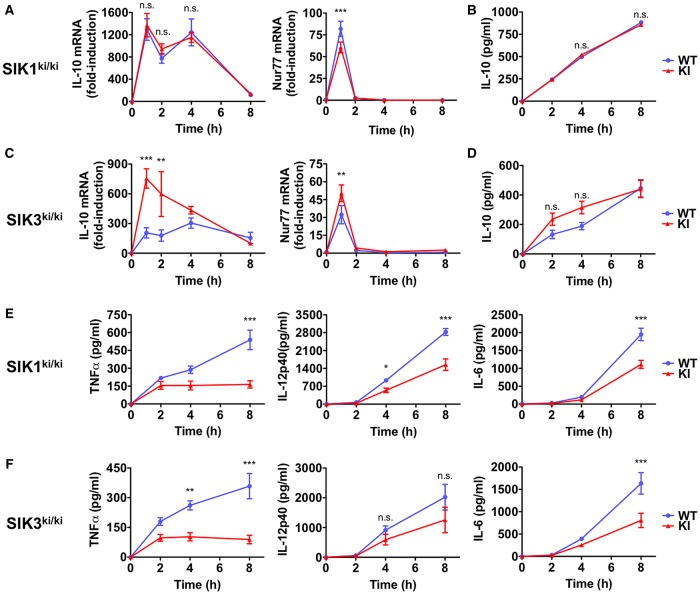
Characterization of macrophages from SIK1 and SIK3 KI mice. (**A**) Kinetics of IL-10 and Nur77 mRNA expression in BMDMs from SIK1-T182A KI mice. Macrophages were stimulated with 100 ng/ml LPS for the indicated times. mRNA levels were measured by qPCR and results are expressed as fold-induction relative to unstimulated WT cells (mean ± SEM, *n* = 4). (**B**) Kinetics of IL-10 secretion from LPS-stimulated BMDMs generated from SIK1-T182A KI mice. Experiment was performed as in (**A**), but the concentration of IL-10 was measured in cell-free culture supernatants using the Bio-Plex Pro Assay System (mean ± SEM, *n* = 4). (**C** and **D**) Experiments performed as in (**A**) and (**B**) using SIK3-T163A KI mice. (**E** and **F**) Kinetics of TNF-α, IL-6 and IL-12p40 secretion from LPS-stimulated BMDMs generated from (**E**) SIK1-T182A and (**F**) SIK3-T163A KI mice. Experiment was performed as in (**B** and **D**), except that the concentrations of TNF-α, IL-6 and IL-12p40 were measured (mean ± SEM, *n* = 4). Statistics represent analysis using two-way ANOVA followed by Bonferroni post-tests.

We therefore characterized the response to LPS in BMDMs from SIK1-T182A and SIK3-T163A mice. The induction of IL-10 and Nur77 mRNA as well as IL-10 secretion were similar between WT and SIK1-T182A macrophages stimulated with LPS (Figure 5A,B). In contrast, LPS-dependent IL-10 and Nur77 mRNA production were raised in macrophages from SIK3-T163A mice in comparison with WT cells (Figure 5C), although not as markedly as in BMDMs from the SIK2-T175A mice. These differences led to a small but not statistically significant increase in IL-10 secretion in macrophages from SIK3-T163A mice (Figure 5D). Despite SIK1-T182A macrophages producing normal levels of IL-10 in response to LPS (Figure 5B), these macrophages produced significantly less TNF-α, IL-12p40 and IL-6 (Figure 5E). Similarly, macrophages from SIK3-T163A mice also secreted less pro-inflammatory cytokines in response to LPS than their WT counterparts (Figure 5F).

Given these results, we investigated the effect of inhibiting SIK2 in combination with either SIK1 or SIK3. For this purpose, we generated SIK1/2 and SIK2/3 double KI mice. Homozygous SIK1/2 double KI mice are viable, fertile and appear indistinguishable from WT animals at the gross anatomical level. In contrast, homozygous SIK2/3 double KI animals are embryonic lethal (0 of 41 animals; expected SIK2/3 double KI ∼10). SIK2/3 double KI embryos were still present at Mendelian ratios at day E15.5 (Supplementary Figure S4), indicating that lethality occurred past this point of gestation. Haematopoiesis is established in embryos by this stage with the haematopoietic stem cells being housed in the foetal liver, which allowed us to generate FLDMs and investigate their phenotype *in vitro*. Although SIK2 remains the most abundant SIK activity in FLDMs, SIK3 makes a greater contribution towards total SIK activity in these cells in comparison with BMDMs (Figure 1B).

**Figure 6.**
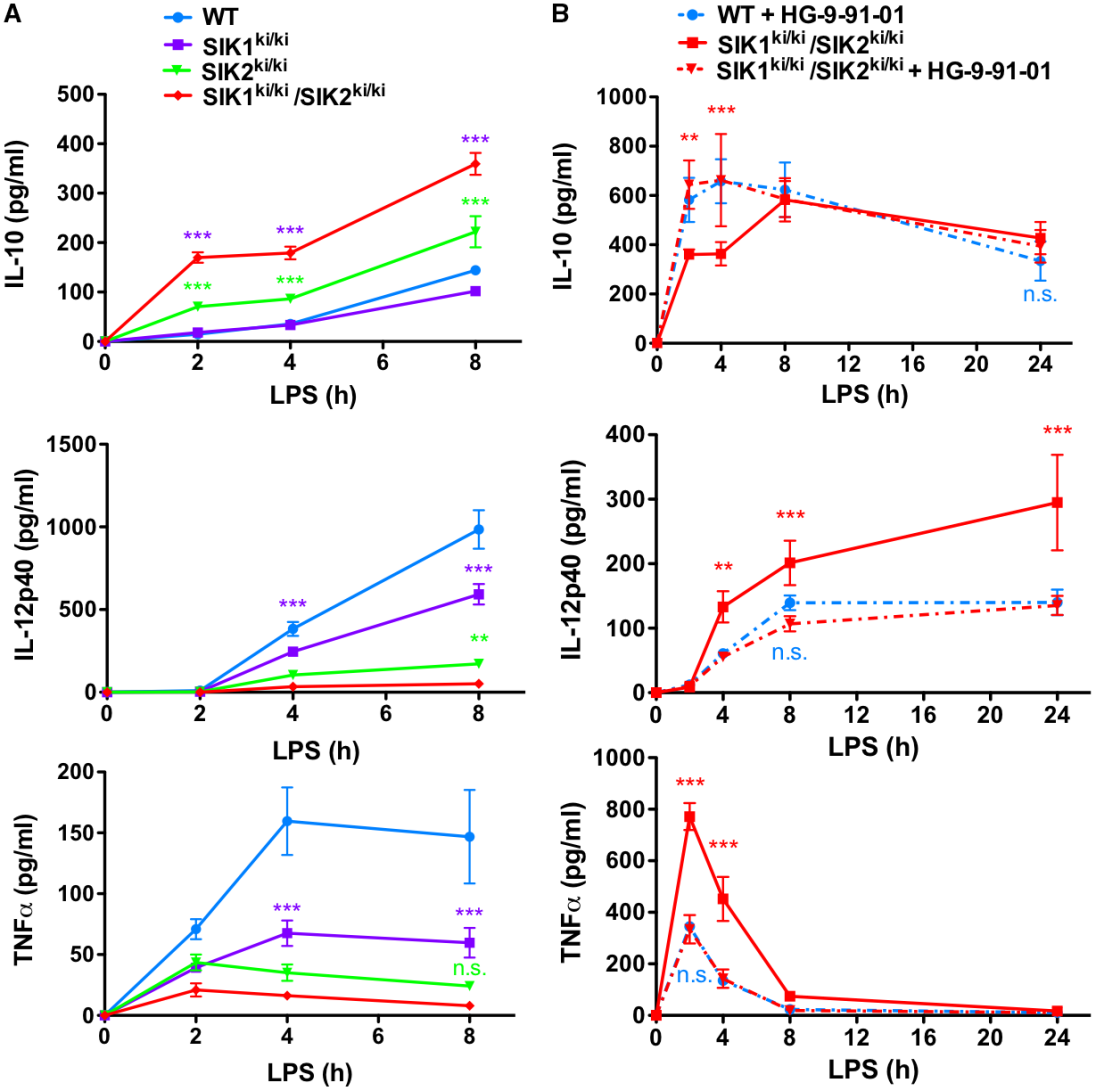
Cytokine production in SIK1/2 double KI macrophages. (**A**) BMDMs from WT (blue), SIK1 KI (purple), SIK2 KI (green) and SIK1/2 double KI mice (red) were stimulated with 100 ng/ml LPS for the indicated times. The concentrations of cytokines in cell-free culture supernatants were measured using the Bio-Plex Pro Assay System (mean ± SEM, *n* = 4). Statistics represent analysis by two-way ANOVA followed by Bonferroni post-tests comparing SIK1/2 double KI with SIK1 KI macrophages (purple asterisks) or SIK2 KI macrophages (green asterisks). (**B**) BMDMs from WT and SIK1/2 double KI mice were treated with vehicle control (solid lines) or 500 nM HG-9-91-01 (dashed lines) for 1 h prior to stimulation with 100 ng/ml LPS for the indicated times. The concentrations of cytokines in cell-free culture supernatants were measured using the Bio-Plex Pro Assay System (mean ± SEM, *n* = 3). Statistics represent analysis by two-way ANOVA followed by Bonferroni post-tests comparing SIK1/2 double KI macrophages treated with HG-9-91-01 to SIK1/2 DKI macrophages treated with vehicle control (red asterisks) or WT macrophages treated with HG-9-91-01 (n.s.).

We found that genetic inactivation of SIK1/2 led to a further increase in IL-10 produced in BMDMs stimulated with LPS when compared with cells in which only SIK1 or SIK2 was inactivated (Figure 6A). Moreover, macrophages from SIK1/2 double KI mice produced higher levels of IL-10 when treated with HG-9-91-01 prior to LPS stimulation, which were equal to those produced by WT cells treated with the same compound (Figure 6B). Similarly, we found a further decrease in the production of IL-12p40 upon inactivation of SIK1 and SIK2 (Figure 6A), and these low levels of cytokine production were further suppressed by HG-9-91-01 (Figure 6B). The production of TNF-α is already low in SIK2 KI macrophages, and the small decrease in TNF-α levels produced in SIK1/2 double KI macrophages was not statistically significant (Figure 6A) but was further suppressed by pretreatment with HG-9-91-01 (Figure 6B). When macrophages were treated with Pam_3_CSK_4_ instead of LPS, a significant effect on cytokine production was observed when mutating both SIK1 and SIK2 in comparison with the individual KI macrophages (Supplementary Figure S5).

**Figure 7.**
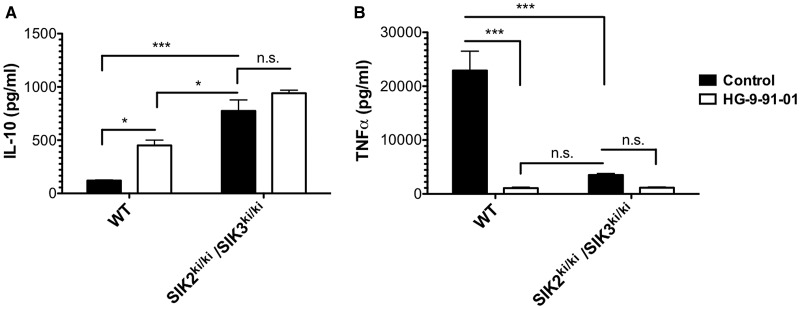
Cytokine production in SIK2/3 double KI macrophages. FLDMs from WT and SIK2/3 double KI mice were treated with vehicle control (white bars) or 500 nM HG-9-91-01 (black bars) for 1 h prior to stimulation with 100 ng/ml LPS for 2–6 h. The concentration of IL-10 (**A**; 2 h) or TNF-α (**B**; 6 h) in cell-free culture supernatants was measured using the Bio-Plex Pro Assay System. Owing to the low yield of macrophages, it was necessary to pool the cells from three foetal livers for the experiment. Error bars represent the SEM for three independent stimulations. Results are representative of three independent experiments (mean ± SEM). Statistics represent analysis by one-way ANOVA, followed by Bonferroni post-tests.

FLDMs from SIK2/3 double KI mice produced large quantities of IL-10 in response to LPS, levels much greater than WT macrophages (Figure 7A). Interestingly, the levels of IL-10 produced by the SIK2/3 double KI macrophages even surpassed those secreted by WT macrophages treated with HG-9-91-01 (Figure 7A). It should be noted that HG-9-91-01 could further elevate IL-10 production slightly in SIK2/3 double KI macrophages, highlighting the contribution of SIK1 to suppression of IL-10 production. Macrophages from SIK2/3 double KI mice produced low levels of TNF-α when compared with WT macrophages (Figure 7B). Collectively, our data indicate that all three isoforms; SIK1, SIK2 and SIK3, contribute towards the overall phenotype of macrophages.

### Differentiation of macrophages in the presence of pan-SIK inhibitors leads to a ‘memory’ response

**Figure 8.**
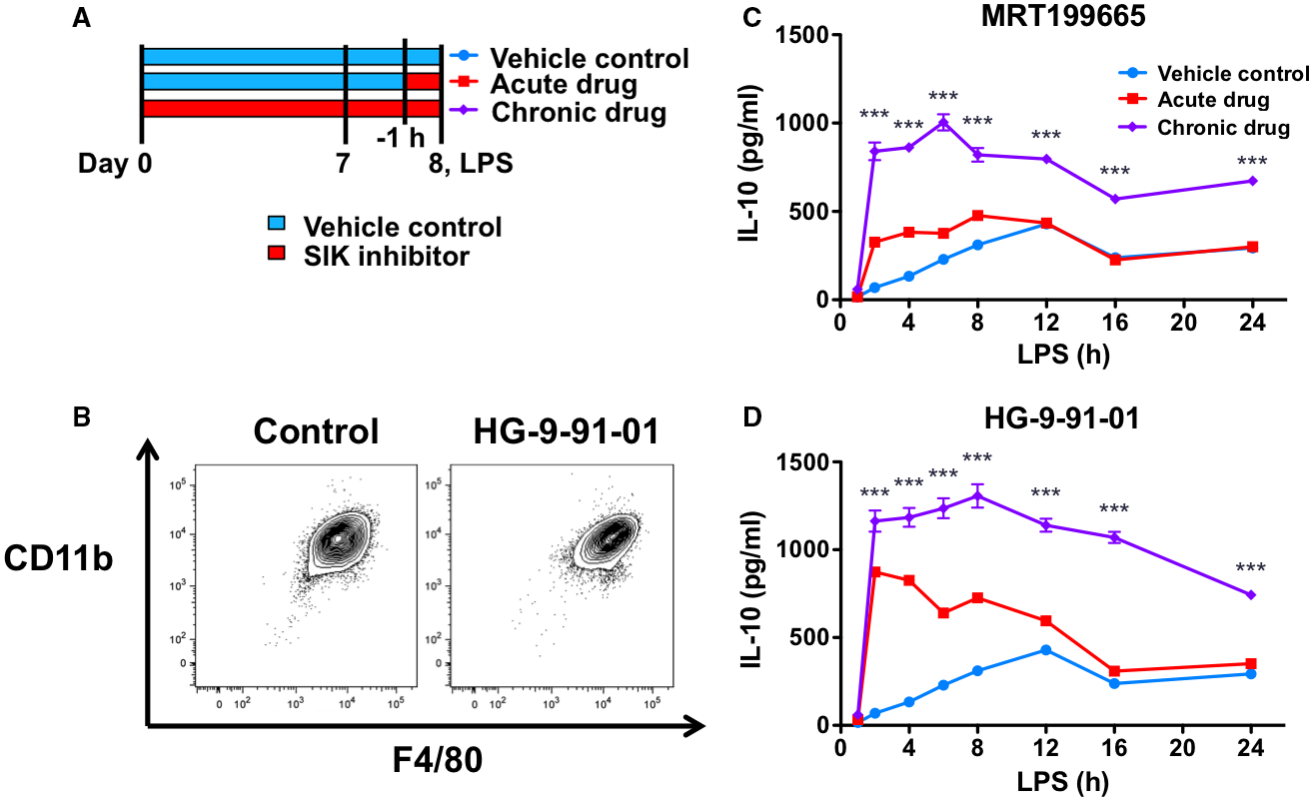
Differentiation of macrophages in the presence of small-molecule inhibitors of the SIKs leads to a population of high IL-10-producing macrophages. (**A**) Schematic diagram depicting the different treatment regimens for the experiment. (**B**) Differentiation of macrophages in the presence of HG-9-91-01 does not affect the proportion of F4/80, CD11b-positive cells. Bone marrow cells were differentiated in the presence of 100 nM HG-9-91-01 or vehicle control for 7 days in M-CSF media. Cells were harvested, stained for F4/80 and CD11b and analyzed by flow cytometry. Results are representative of two independent experiments each performed using independent macrophage cultures from four mice. (**C** and **D**) Differentiation of macrophages in the presence of (**C**) MRT199665 and (**D**) HG-9-91-01 results in the production of high levels of IL-10. Bone marrow cells were differentiated in the presence of vehicle control (blue/red lines) or 125 nM MRT199665 (purple line, **C**) or 100 nM HG-9-91-01 (purple line, **D**) in L929-conditioned media as a source of M-CSF. On day 7, macrophages were harvested and either re-plated in the presence of vehicle control (blue/red lines) or retained in the drug (purple line). Twenty-four hours later, macrophages were treated with vehicle control (blue/purple lines) or 125 nM MRT199665 (red line, **C**) or 100 nM HG-9-91-01 (red line, **D**) for 1 h. All cell populations were then stimulated with 100 ng/ml LPS for the indicated times, and the concentration of IL-10 in cell-free culture supernatants was measured by ELISA. Results were generated using cells pooled from three mice. Error bars represent the SEM for three independent stimulations. The data are representative of two (**C**) or three (**D**) independent experiments. Statistics represent analysis using two-way ANOVA and Bonferroni post-tests comparing the results obtained with acute and chronic drug treatments.

In previous experiments, pharmacological inhibitors of SIKs were added to the culture media of fully differentiated macrophages 1 h prior to stimulation with TLR ligands. In contrast, cells from the KI mice would be differentiating in the context of reduced SIK activity. Prompted by our results in SIK2/3 double KI macrophages, we investigated the effect of adding HG-9-91-01 or MRT199665 to the culture medium during the differentiation of macrophages on IL-10 production (Figure 8A). Following 7 days in culture in media containing M-CSF, the presence of HG-9-91-01 did not affect the expression of the macrophage markers CD11b or F4/80 (Figure 8B). Consistent with previous results, the addition of HG-9-91-01 or MRT199665 just prior to stimulation with LPS led to an accelerated production of IL-10 relative to cells that did not receive the inhibitor. IL-10 levels between these conditions converged upon 12–24 h of LPS treatment (Figure 8C,D). In contrast, we found that macrophages differentiated in the presence of either HG-9-91-01 or MRT199665 produced more IL-10, and this difference was still sustained following 24 h LPS stimulation (Figure 8C,D). These results indicate that inhibition of the SIKs during the differentiation of macrophages further potentiates IL-10 production, thereby explaining the observation in the SIK2/3 double KI macrophages (Figure 7A).

**Figure 9.**
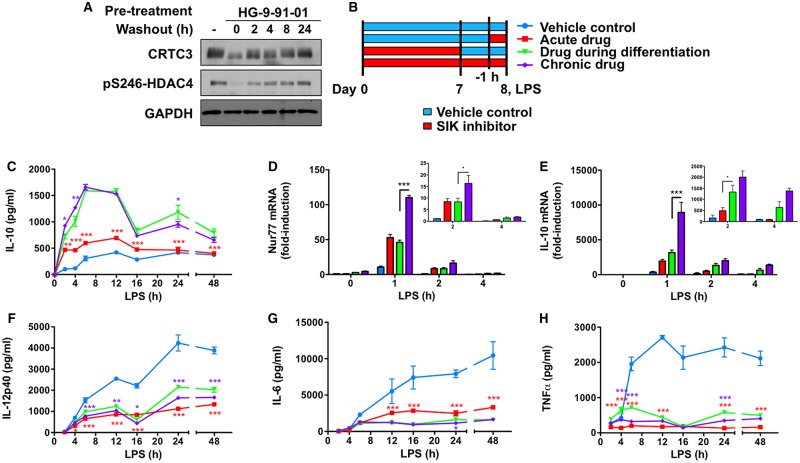
Macrophages differentiated in the presence of SIK inhibitors have a stable pro-resolution M2-like phenotype. (**A**) Kinetics of phosphorylation of CRTC3 and HDAC4 after removal of HG-9-91-01. BMDMs were left untreated (−) or treated with 500 nM HG-9-91-01 for 1 h to induce the dephosphorylation of SIK substrates. The cells were subsequently washed with fresh media and lysed at the indicated times following the removal of the inhibitor. Cell extracts were immunoblotted with the indicated antibodies. Results are representative of two independent experiments. (**B**) Schematic diagram depicting the different treatment regimens for the experiment. (**C–H**) Bone marrow cells were differentiated in the presence of vehicle control (blue/red lines) or 100 nM HG-9-91-01 (green/purple lines) in media containing M-CSF. On day 7, macrophages were harvested and either replated in the presence of vehicle control (blue/red/green lines) or retained in 100 nM HG-9-91-01 (purple line). Twenty-four hours later, macrophages were treated with vehicle control (blue/green/purple lines) or 100 nM HG-9-91-01 (red line) for 1 h. All cell populations were then stimulated with 100 ng/ml LPS for the indicated times. The concentrations of IL-10 (**C**), IL-12p40 (**F**), IL-6 (**G**) and TNF-α (**H**) in cell-free culture supernatants were measured using the Bio-plex Pro Assay System. The relative levels of Nur77 (**D**) and IL-10 (**E**) mRNA were determined by qPCR. qPCR results are expressed as fold-induction relative to control cells that were not treated with drug or LPS. Results were generated using cells pooled from four mice. Error bars represent the SEM for three independent stimulations. Data are representative of three independent experiments. Statistics represent analysis by two-way ANOVA followed by Bonferroni post-tests comparing drug treatment during differentiation only with acute drug (red asterisks) or chronic drug (purple asterisks).

An advantage of using small-molecule ATP-competitive SIK inhibitors is that their actions are reversible. Indeed, phosphorylation of physiological substrates of the SIKs, including CRTC3 and HDAC4, rapidly returns to normal levels after HG-9-91-01 has been removed from the culture medium (Figure 9A). Interestingly, macrophages differentiated in the presence of HG-9-91-01 continued to produce very high levels of IL-10 even after the compound had been removed from the culture media (Figure 9B,C). Although Nur77 mRNA levels were still elevated in LPS-stimulated macrophages upon removal of HG-9-91-01, its presence was required for maximal Nur77 expression (Figure 9D). At early time points post-LPS stimulation, IL-10 mRNA levels were elevated in macrophages treated with HG-9-91-01 regardless of the treatment regimen (Figure 9E). However, there was a prolonged increase in IL-10 mRNA levels at late time points (2 and 4 h) when macrophages had been differentiated in the presence of HG-9-91-01, and this increase was still detected when the drug was removed from the culture medium prior to stimulation (Figure 9E). These data suggest that the regulation of IL-10 secretion by the SIKs is mediated by CREB-dependent and -independent pathways, with the latter being revealed as a functional consequence of SIK activity during the differentiation of macrophages.

Differentiation of macrophages in the presence of HG-9-91-01 also had dramatic effects on other markers of pro-resolution M2-like macrophages. The production of IL-12p40 (Figure 9F), IL-6 (Figure 9G) and TNF-α (Figure 9H) was suppressed in macrophages differentiated in the presence of HG-9-91-01 even after removal of the drug. Similarly, the expression of Arg1 and LIGHT following LPS stimulation was higher in cells differentiated in the presence of HG-9-91-01 compared with acute treatment of cells with the drug. SPHK1 mRNA levels were similar between macrophages treated acutely or chronically with HG-9-91-01 (Supplementary Figure S6). Our data indicate that inhibition of SIK1, SIK2 and SIK3 during differentiation leads to a stable anti-inflammatory phenotype in mouse macrophages.

## Discussion

Pro-resolution M2-like macrophages play a key role in driving the resolution of inflammation. Therapeutic approaches aimed at accentuating the numbers of this macrophage population at sites of inflammation could provide long-term disease-modifying benefits to patients suffering from chronic inflammatory and autoimmune diseases such as rheumatoid arthritis. Our research has highlighted that one potential strategy to achieve this goal involves the inhibition of the SIKs [17,23]. To address the role of SIK1, SIK2 and SIK3 in controlling macrophage polarization, we generated KI mice in which we introduced mutations that render each SIK isoform catalytically inactive. This genetic approach allows us to specifically analyze the (patho)physiological role of the enzymatic activity of these protein kinases. Targeted kinase inactivation is the preferred approach when assessing new therapeutic strategies that would eventually rely on pharmacological methods to inhibit the protein kinase. The phenotypes in mouse knock-out (KO) models are attributed to the combination of the functionalities of all the domains in the protein. One major difference between the two genetic strategies is that the loss of the protein in the mouse KO can significantly alter the composition of complexes of related proteins, as the protein is no longer available to bind to its endogenous-binding partners. In contrast, the assembly of the endogenous protein complexes can still take place in the mouse KI. This major difference can therefore lead to discrepancies in the phenotypes observed in KO and KI mice [28]. The novel mouse lines generated as part of our study complement currently available mouse KO strains [29–31] to assess the physiological roles of the SIKs and the therapeutic potential of targeting this group of enzymes.

Mouse macrophages express all three isoforms of the SIKs but at varying levels. Consistent with SIK2 being the predominant isoform as judged by kinase activity, macrophages from SIK2-T175A mice present several features associated with pro-resolution M2-like macrophages, including enhanced CREB-dependent gene transcription, increased secretion of IL-10, decreased production of the pro-inflammatory cytokines IL-6, IL-12p40 and TNF-α and enhanced expression of markers including Arg1, LIGHT and SPHK1. In contrast, macrophages from SIK1-T182A and SIK3-T163A mice displayed a smaller spectrum of characteristics associated with pro-resolution M2-like macrophages and they were less prominent than observed in SIK2-T175A macrophages. Notably, genetic inactivation of SIK1 or SIK3 had little effect on IL-10 production while having a significant impact on the production of pro-inflammatory cytokines in response to LPS. Although the data indicate that SIK2 is a major isoform controlling macrophage polarization, inhibition of SIK1 and SIK3 is also required to fully polarize macrophages towards a pro-resolution M2-like phenotype. This was consistent with the observation that the pan-SIK inhibitor HG-9-91-01 could further promote the features of pro-resolution M2-like macrophages in cells from SIK2-T175A mice. Moreover, genetic inactivation of SIK1 or SIK3 in SIK2-T175A macrophages enhanced the M2-like phenotype of these cells. Our results suggest that inhibition of all three SIK isoforms is required to fully polarize macrophages towards the pro-resolution M2-like phenotype in BMDMs and FLDMs. Importantly, our data provide genetic validation that inhibition of the enzymatic activity of the SIKs leads to the formation of an anti-inflammatory, pro-resolution macrophage phenotype.

The molecular mechanisms by which the SIKs control cytokine production in macrophages are complex, and the relative contribution of each SIK isoform appears to differ between cytokines. One striking example is the regulation of IL-6 whereby the role of the SIKs differs in a temporal manner. Intriguingly, IL-6 has a putative CREB-binding site in its promoter region, consistent with its cAMP-dependent transcription following treatment with agents such as PGE_2_ [12,32]. Thus, at early time points, the SIKs may suppress IL-6 production through the control of CREB-dependent transcriptional activation. In contrast, at late time points, the SIKs promote IL-6 production through a poorly defined, CREB-independent mechanism. Our results are consistent with other reports showing that the effect of SIK inhibition on pro-inflammatory cytokines, including IL-6, operates via pathways independent of IL-10 [12,21].

Investigations in several laboratories around the world have demonstrated that inhibition of the SIKs induces an anti-inflammatory phenotype in mouse and human macrophages [12,17,21–23,33]. Surprisingly, Sanosaka et al. [34] found that SIK3 KO mice were more susceptible to septic shock than WT mice, which correlated with an enhanced pro-inflammatory phenotype in thioglycolate-elicited peritoneal macrophages. In particular, SIK3 KO thioglycolate-elicited peritoneal macrophages produced higher levels of IL-6 and nitric oxide (NO) in response to LPS [34]. In our studies, we found that BMDMs from SIK3-T163A mice secreted lower levels of pro-inflammatory cytokines, including IL-6 and TNF-α, in response to LPS. Consistent with the experiments using SIK3 KO macrophages [34], we found that the induction of iNOS mRNA and protein was accelerated in FLDMs from SIK3-T163A mice in response to LPS (Supplementary Figure S7B,C). However, NO production was not significantly affected by genetic inactivation of SIK3 (Supplementary Figure S7D). Moreover, macrophages treated with the pan-SIK inhibitor HG-9-91-01 produced either slightly less or similar amounts of NO in response to LPS (Supplementary Figure S7E–H). SIK inhibition also had little effect on NO production in macrophages further polarized by the addition of IFN-γ along with LPS (Supplementary Figure S7I). Collectively, the results suggest that the loss of the entire SIK3 protein during differentiation may influence macrophage function in ways that are independent of its catalytic activity. Alternatively, the various isoforms of the SIKs may have distinct functions in different macrophage populations. Consistent with this hypothesis, we found that the amount of SIK2 and SIK3 kinase activity was higher in FLDMs in comparison with BMDMs. Given the potential for environmental effects on the immune system, for instance through the modulation of the gut microbiome [35], it will be important to perform side-by-side comparisons at the same institution to address these differences between the two mouse models of SIK3 function.

Excitingly, we discovered that inhibition of the SIKs during macrophage differentiation leads to a stable anti-inflammatory phenotype. Macrophages continued to produce high levels of IL-10 and low levels of pro-inflammatory cytokines even after removal of HG-9-91-01 or MRT199665 (data not shown) from the culture medium. Although the molecular mechanism remains undefined, we speculate that this stable phenotype is the result of epigenetic changes occurring within transcriptional control elements of genes encoding cytokines. This suggestion is not without precedent since innate immune memory has been attributed to global epigenetic changes within myeloid cells [36]. Inhibition of the SIKs allows the dephosphorylation of class IIa HDACs such as HDAC4 [21], which may affect histone modifications at cytokine promoters. As epigenetic marks would remain even after reactivation of the SIKs and inactivation of CRTC3 and HDAC4, through phosphorylation-dependent 14-3-3 interactions, this may explain the effects of long-term SIK inhibition. Consistent with this hypothesis, we found that the return of IL-10 mRNA back to basal levels was delayed when macrophages had been differentiated in the presence of HG-9-91-01.

It is tempting to speculate about the potential implications of our findings on drug therapy in the future. One immediate consequence of macrophages remembering the sustained inhibition of the SIKs long after these enzymes have been reactivated is the potential for a drug ‘holiday’. The regimen for drug administration could include a short period where the patient stops taking a SIK inhibitor. This may limit the on-target and off-target side effects associated with any future medication. It will therefore be important to compare the development of inflammation in mouse models of disease using various therapeutic regimens once highly specific compounds with optimal drug metabolism and pharmcokinetic properties have been developed.

The developmental phenotypes of the various mouse KI lines indicate that SIK1, SIK2 and SIK3 also have distinct but overlapping roles in different tissues. We hypothesize that, in certain cases, the relative importance of SIK1, SIK2 and SIK3 is simply the result of the expression levels of each isoform in different tissues, while in other circumstances, each isoform has different roles potentially guided by the divergent C-terminal tails of SIK1, SIK2 and SIK3. SIK3 is the most abundant isoform expressed in embryos at day E16.5 as judged by kinase activity (Supplementary Figure S8) and its inactivation results in fewer and smaller live animals with skeletal defects at weaning (3–4 weeks). This phenotype is reminiscent of the SIK3 KO mouse [29]. Indeed, for both models, only ∼40% of SIK3 homozygous KI or KO animals reached weaning (Supplementary Figure S2) [37]. Conversely, genetic inactivation of both SIK1 and SIK2 had no effect on mouse development. Thus, SIK3 activity is required and sufficient for normal mouse development. Additional inactivation of SIK2 resulted in no viable SIK2/3 double KI mice at weaning (data not shown). Of the animals that die before weaning, the SIK3 KO mice die after the first day post-birth [37]; while the time point of death between day E15.5 and weaning of the SIK3 KI and SIK2/3 double KI mice remains to be defined. The consequence of inhibition of the catalytic activity of the SIKs in adult animals awaits the generation of conditional KI animals and/or the development of highly potent and selective small-molecule inhibitors.

In conclusion, we demonstrate that all three isoforms of the SIKs contribute towards macrophage biology. Our results indicate that inhibiting SIK2 and SIK3 in mature macrophages induces a population of macrophages producing high levels of IL-10 and low levels of pro-inflammatory cytokines including TNF-α. Notably, inhibition of SIK2 and SIK3 during the differentiation of macrophages leads to a pronounced and stable anti-inflammatory macrophage population. Macrophages differentiated in the presence of HG-9-91-01 or MRT199665 continue to produce high levels of IL-10 and low levels of pro-inflammatory cytokines even after withdrawal of the drug. We therefore propose that pan-SIK inhibitors would be the most effective therapeutic strategy. Furthermore, the dosing regimen may allow for drug holidays as removal of the SIK inhibitor to allow the enzymes to return to full activity did not impede the anti-inflammatory phenotype of the macrophages generated in the presence of the drug. This therapeutic approach could provide a set of multifactorial benefits including a reduction in on-target and off-target side effects by the new drugs.

## Abbreviations

Arg1: arginase 1
BMDMs: bone marrow-derived macrophages
cAMP: cyclic AMP
CREB: cAMP response element-binding protein
CRTC: CREB-regulated transcription co-activator
DUSP: dual specificity phosphatase
ES: embryonic stem
FCS: foetal calf serum
FLDM: foetal liver-derived macrophages
GAPDH: glyceraldehyde 3-phosphate dehydrogenase
HDAC: histone deacetylase
iNOS: inducible NOS
IFN-γ: interferon γ
IL: interleukin
IL-1ra: IL-1 receptor antagonist
IRF: interferon regulatory factor
KI: knock-in
KO: knock-out
LIGHT: TNF ligand superfamily member 14
LPS: lipopolysaccharide
M-CSF: macrophage colony-stimulating factor
MSK: mitogen- and stress-activated protein kinase
NF-κB: nuclear factor κB
PGE_2_: prostaglandin E_2_
SIK: salt-inducible kinase
SPHK1: sphingosine kinase 1
STAT: signal transducer and activator of transcription
TLR: Toll-like receptor
TNF: tumour necrosis factor
WT: wild type.

## References

[1] Mosser D.M. and Edwards J.P. (2008) Exploring the full spectrum of macrophage activation. Nat. Rev. Immunol. 8, 958–969 doi:10.1038/nri2448 19029990 PMC2724991

[2] Sica A. and Mantovani A. (2012) Macrophage plasticity and polarization: in vivo veritas. J. Clin. Invest. 122, 787–795 doi:10.1172/JCI59643 22378047 PMC3287223

[3] Martinez F.O. and Gordon S. (2014) The M1 and M2 paradigm of macrophage activation: time for reassessment. F1000Prime Rep. 6, 13 doi:10.12703/P6-13 24669294 PMC3944738

[4] Fleming B.D. and Mosser D.M. (2011) Regulatory macrophages: setting the threshold for therapy. Eur. J. Immunol. 41, 2498–2502 doi:10.1002/eji.201141717 21952805 PMC4299459

[5] Murray P.J., Allen J.E., Biswas S.K., Fisher E.A., Gilroy D.W., Goerdt S. et al. (2014) Macrophage activation and polarization: nomenclature and experimental guidelines. Immunity 41, 14–20 doi:10.1016/j.immuni.2014.06.008 25035950 PMC4123412

[6] Fleming B.D., Chandrasekaran P., Dillon L.A., Dalby E., Suresh R., Sarkar A. et al. (2015) The generation of macrophages with anti-inflammatory activity in the absence of STAT6 signaling. J. Leukoc. Biol. 98, 395–407 doi:10.1189/jlb.2A1114-560R 26048978 PMC4541501

[7] McInnes I.B. and Schett G. (2011) The pathogenesis of rheumatoid arthritis. N. Engl. J. Med. 365, 2205–2219 doi:10.1056/NEJMra1004965 22150039

[8] Li J., Hsu H.-C. and Mountz J.D. (2012) Managing macrophages in rheumatoid arthritis by reform or removal. Curr. Rheumatol. Rep. 14, 445–454 doi:10.1007/s11926-012-0272-4 22855296 PMC3638732

[9] Tall A.R. and Yvan-Charvet L. (2015) Cholesterol, inflammation and innate immunity. Nat. Rev. Immunol. 15, 104–116 doi:10.1038/nri3793 25614320 PMC4669071

[10] Clark K. (2014) Protein kinase networks that limit TLR signalling. Biochem. Soc. Trans. 42, 11–24 doi:10.1042/BST20130124 24450622

[11] Hu X., Paik P.K., Chen J., Yarilina A., Kockeritz L., Lu T.T. et al. (2006) IFN-γ suppresses IL-10 production and synergizes with TLR2 by regulating GSK3 and CREB/AP-1 proteins. Immunity 24, 563–574 doi:10.1016/j.immuni.2006.02.014 16713974

[12] MacKenzie K.F., Clark K., Naqvi S., McGuire V.A., Noehren G., Kristariyanto Y. et al. (2013) PGE2 induces macrophage IL-10 production and a regulatory-like phenotype via a protein kinase A-SIK-CRTC3 pathway. J. Immunol. 190, 565–577 doi:10.4049/jimmunol.1202462 23241891 PMC3620524

[13] Ananieva O., Darragh J., Johansen C., Carr J.M., McIlrath J., Park J.M. et al. (2008) The kinases MSK1 and MSK2 act as negative regulators of Toll-like receptor signaling. Nat. Immunol. 9, 1028–1036 doi:10.1038/ni.1644 18690222

[14] Naqvi S., Martin K.J. and Arthur J.S. (2014) CREB phosphorylation at Ser133 regulates transcription via distinct mechanisms downstream of cAMP and MAPK signalling. Biochem. J. 458, 469–479 doi:10.1042/BJ20131115 24438093

[15] Altarejos J.Y. and Montminy M. (2011) CREB and the CRTC co-activators: sensors for hormonal and metabolic signals. Nat. Rev. Mol. Cell. Biol. 12, 141–151 doi:10.1038/nrm3072 21346730 PMC4324555

[16] Screaton R.A., Conkright M.D., Katoh Y., Best J.L., Canettieri G., Jeffries S. et al. (2004) The CREB coactivator TORC2 functions as a calcium- and cAMP-sensitive coincidence detector. Cell 119, 61–74 doi:10.1016/j.cell.2004.09.015 15454081

[17] Clark K., MacKenzie K.F., Petkevicius K., Kristariyanto Y., Zhang J., Choi H.G. et al. (2012) Phosphorylation of CRTC3 by the salt-inducible kinases controls the interconversion of classically activated and regulatory macrophages. Proc. Natl Acad. Sci. U.S.A. 109, 16986–16991 doi:10.1073/pnas.1215450109 23033494 PMC3479463

[18] Cohen P. (2014) The TLR and IL-1 signalling network at a glance. J. Cell Sci. 127, 2383–2390 doi:10.1242/jcs.149831 24829146 PMC4038938

[19] Chen L.-F., Mu Y. and Greene W.C. (2002) Acetylation of RelA at discrete sites regulates distinct nuclear functions of NF-κB. EMBO J. 21, 6539–6548 doi:10.1093/emboj/cdf660 12456660 PMC136963

[20] Berdeaux R., Goebel N., Banaszynski L., Takemori H., Wandless T., Shelton G.D. et al. (2007) SIK1 is a class II HDAC kinase that promotes survival of skeletal myocytes. Nat. Med. 13, 597–603 doi:10.1038/nm1573 17468767

[21] Luan B., Goodarzi M.O., Phillips N.G., Guo X., Chen Y.-D., Yao J. et al. (2014) Leptin-mediated increases in catecholamine signaling reduce adipose tissue inflammation via activation of macrophage HDAC4. Cell Metab. 19, 1058–1065 doi:10.1016/j.cmet.2014.03.024 24768298 PMC4207085

[22] Sundberg T.B., Choi H.G., Song J.-H., Russell C.N., Hussain M.M., Graham D.B. et al. (2014) Small-molecule screening identifies inhibition of salt-inducible kinases as a therapeutic strategy to enhance immunoregulatory functions of dendritic cells. Proc. Natl Acad. Sci. U.S.A. 111, 12468–12473 doi:10.1073/pnas.1412308111 25114223 PMC4151730

[23] Ozanne J., Prescott A.R. and Clark K. (2015) The clinically approved drugs dasatinib and bosutinib induce anti-inflammatory macrophages by inhibiting the salt-inducible kinases. Biochem. J. 465, 271–279 doi:10.1042/BJ20141165 25351958 PMC4286194

[24] Lizcano J.M., Göransson O., Toth R., Deak M., Morrice N.A., Boudeau J. et al. (2004) LKB1 is a master kinase that activates 13 kinases of the AMPK subfamily, including MARK/PAR-1. EMBO J. 23, 833–843 doi:10.1038/sj.emboj.7600110 14976552 PMC381014

[25] Pfaffl M.W. (2001) A new mathematical model for relative quantification in real-time RT-PCR. Nucleic Acids Res. 29, e45 doi:10.1093/nar/29.9.e45 11328886 PMC55695

[26] Horike N., Takemori H., Katoh Y., Doi J., Min L., Asano T. et al. (2003) Adipose-specific expression, phosphorylation of Ser794 in insulin receptor substrate-1, and activation in diabetic animals of salt-inducible kinase-2. J. Biol. Chem. 278, 18440–18447 doi:10.1074/jbc.M211770200 12624099

[27] Saraiva M. and O'Garra A. (2010) The regulation of IL-10 production by immune cells. Nat. Rev. Immunol. 10, 170–181 doi:10.1038/nri2711 20154735

[28] Pauls E., Nanda S.K., Smith H., Toth R., Arthur J.S. and Cohen P. (2013) Two phases of inflammatory mediator production defined by the study of IRAK2 and IRAK1 knock-in mice. J. Immunol. 191, 2717–2730 doi:10.4049/jimmunol.1203268 23918981 PMC3849919

[29] Sasagawa S., Takemori H., Uebi T., Ikegami D., Hiramatsu K., Ikegawa S. et al. (2012) SIK3 is essential for chondrocyte hypertrophy during skeletal development in mice. Development 139, 1153–1163 doi:10.1242/dev.072652 22318228

[30] Sasaki T., Takemori H., Yagita Y., Terasaki Y., Uebi T., Horike N. et al. (2011) SIK2 is a key regulator for neuronal survival after ischemia via TORC1-CREB. Neuron 69, 106–119 doi:10.1016/j.neuron.2010.12.004 21220102

[31] Stenström K., Takemori H., Bianchi G., Katz A.I. and Bertorello A.M. (2009) Blocking the salt-inducible kinase 1 network prevents the increases in cell sodium transport caused by a hypertension-linked mutation in human alpha-adducin. J. Hypertens. 27, 2452–2457 doi:10.1097/HJH.0b013e328330cf15 19657284

[32] Dendorfer U., Oettgen P. and Libermann T.A. (1994) Multiple regulatory elements in the interleukin-6 gene mediate induction by prostaglandins, cyclic AMP, and lipopolysaccharide. Mol. Cell Biol. 14, 4443–4454 doi:10.1128/MCB.14.7.4443 8007951 PMC358816

[33] Lombardi M.S., Gilliéron C., Dietrich D. and Gabay C. (2016) SIK inhibition in human myeloid cells modulates TLR and IL-1R signaling and induces an anti-inflammatory phenotype. J. Leukoc. Biol. 99, 711–721 doi:10.1189/jlb.2A0715-307R 26590148

[34] Sanosaka M., Fujimoto M., Ohkawara T., Nagatake T., Itoh Y., Kagawa M. et al. (2015) Salt-inducible kinase 3 deficiency exacerbates lipopolysaccharide-induced endotoxin shock accompanied by increased levels of pro-inflammatory molecules in mice. Immunology 145, 268–278 doi:10.1111/imm.12445 25619259 PMC4427391

[35] Beura L.K., Hamilton S.E., Bi K., Schenkel J.M., Odumade O.A., Casey K.A. et al. (2016) Normalizing the environment recapitulates adult human immune traits in laboratory mice. Nature 532, 512–516 doi:10.1038/nature17655 27096360 PMC4871315

[36] Saeed S., Quintin J., Kerstens H.H., Rao N.A., Aghajanirefah A., Matarese F. et al. (2014) Epigenetic programming of monocyte-to-macrophage differentiation and trained innate immunity. Science 345, 1251086 doi:10.1126/science.1251086 25258085 PMC4242194

[37] Uebi T., Itoh Y., Hatano O., Kumagai A., Sanosaka M., Sasaki T. et al. (2012) Involvement of SIK3 in glucose and lipid homeostasis in mice. PLoS ONE 7, e37803 doi:10.1371/journal.pone.0037803 22662228 PMC3360605

